# Children Who Received PCV-10 Vaccine from a Two-Dose Vial without Preservative Are Not More Likely to Develop Injection Site Abscess Compared with Those Who Received Pentavalent (DPT-HepB-Hib) Vaccine: A Longitudinal Multi-Site Study

**DOI:** 10.1371/journal.pone.0097376

**Published:** 2014-06-04

**Authors:** Yemane Berhane, Alemayehu Worku, Meaza Demissie, Neghist Tesfaye, Nega Asefa, Worku Aniemaw, Berhe Weldearegawi, Yigzaw Kebede, Tigist Shiferaw, Amare Worku, Lemessa Olijira, Behailu Merdekios, Yemane Ashebir, Takele Tadesse, Yadeta Dessie, Solomon Meseret, Gestane Ayele

**Affiliations:** 1 Addis Continental Institute of Public Health, Addis Ababa, Ethiopia; 2 Federal Ministry of Health, Addis Ababa, Ethiopia; 3 College of Health Sciences, Haramaya University, Harar, Ethiopia; 4 College of Medicine and Health Sciences, Arbaminch University, Arbaminch, Ethiopia; 5 Department of Public Health, Mekelle University, Mekelle, Ethiopia; 6 Institute of Public Health, College of Medicine and Health Sciences, University of Gondar Gondar, Ethiopia; National Taiwan University Hospital, Taiwan

## Abstract

**Background:**

The single dose pneumonia ten-valent vaccine has been widely used and is highly efficacious against selected strains *Streptococcus pneumonia.* A two-dose vial without preservative is being introduced in developing countries to reduce the cost of the vaccine. In routine settings improper immunization practice could result in microbial contamination leading to adverse events following immunization.

**Objective:**

To monitor adverse events following immunization recommended for routine administration during infancy by comparing the rate of injection-site abscess between children who received PCV-10 vaccine and children who received the Pentavalent (DPT-HepB-Hib) vaccine.

**Methods:**

A longitudinal population-based multi-site observational study was conducted between September 2011 and October 2012. The study was conducted in four existing Health and Demographic Surveillance sites run by public universities of Abraminch, Haramaya, Gondar and Mekelle. Adverse events following Immunization were monitored by trained data collectors. Children were identified at the time of vaccination and followed at home at 48 hour and 7 day following immunization. Incidence of abscess and relative risk with the corresponding 95% Confidence Intervals were calculated to examine the risk difference in the comparison groups.

**Results:**

A total of 55, 268 PCV and 37, 480 Pentavalent (DPT-HepB-Hib) vaccinations were observed. A total of 19 adverse events following immunization, 10 abscesses and 9 deaths, were observed during the one year study period. The risk of developing abscess was not statistically different between children who received PCV-10 vaccine and those received Pentavalent (RR = 2.7, 95% CI 0.576–12.770), and between children who received the first aliquot of PCV and those received the second aliquot of PCV (RR = 1.72, 95% CI 0.485–6.091).

**Conclusion:**

No significant increase in the risk of injection site abscess was observed between the injection sites of PCV-10 vaccine from a two-dose vial without preservative and pentavalent (DPT-HepB-Hib) vaccine in the first 7 days following vaccination.

## Introduction

PCV-10 is a 10-valent Pneumococcal Conjugate Vaccine active against pneumococcal serotypes 1, 4, 5, 6B, 7F, 9V, 14, 18C, 19F, and 23F. It is manufactured by GlaxoSmithKline Biologicals (GSK) to protect children against invasive pneumococcal disease (IPD) and pneumococcal acute otitis media (AOM) caused by serotypes included in the vaccine. PCV-10 vaccine has been shown to be safe and does not cause adverse events beyond what is common with current childhood vaccines [1]–[4].

Vaccines offered in multi-dose presentations can reduce the cold-chain capacity requirements and medical waste handling needs for introduction of new vaccines, as well as decrease the cost per dose, but may pose potential safety concerns that are avoidable with immediately disposable single-dose formats [5]. Multi-dose vaccines have traditionally contained a preservative to minimize the risk of contamination, but there have also been public safety concerns raised about preservatives [6]. A two-dose preservative-free vial presentation can reduce the programmatic capacity requirements related to the introduction of a new vaccine while addressing the public safety concerns with regard to preservatives. Non-sterile injection practice or use of a two-dose preservative-free vial after the recommended amount of time (6 hours) from opening the vial could result in unsafe injections.

Pharmacovigilance is an important mechanism for evaluating new vaccines globally and most suspected Adverse Events Following Immunizations (AEFIs) are reported from passive safety surveillance systems in developed countries. For developing countries, WHO has provided guidelines for establishing immunization safety surveillance [7], but WHO estimates that at least 65% of the world’s populations live in countries still lacking a functional pharmacovigilance system [8]. Thus potential AEFIs may not be detected through routine surveillance systems in developing countries. Thus, designing a mechanism for evaluating and proper monitoring of AEFIs is, therefore, very key in the introduction of new vaccines.

The low incidence of most AEFIs and the low specificity of existing disease detection systems also make identification and interpretation of suspected AEFIs challenging. For example, there are limited data available that describe the baseline incidence of injection-site abscesses. In a US passive AEFI surveillance system reporting on >1–9 billion net doses of human vaccine, the risk of adverse event reporting was 11.4/100,000 immunizations. Commonly reported adverse events included fever (in 25.8% of reports), injection site hypersensitivity (in 15.8%), injection site oedema (in 10.8%) and vasodilation (in 10.5%); 426 cases of injection-site abscesses were identified which comprised 0.3% of all adverse event reports suggesting a risk of 3.4 per 10 million immunizations [9]. Other safety studies for yellow fever vaccination and Bacille Calmette-Guérin (BCG) vaccination in Ivory Coast and Australia also reported low rates of injection-site abscesses [10].

This study was conducted to examine whether the use of a two-dose preservative-free vaccine given in the routine immunization program in Ethiopia is associated with excess adverse event following immunization. The two-dose preservative-free vaccine was given at the same time Pentavalent vaccine is given to children under the age of one year. The hypothesis is that if correct vaccination procedures are followed, the risk of injection-site abscess would show no statistically significant difference between children received the preservative-free PCV-10 vaccine and a fully liquid one dose vial Pentavalent (DTP-Hib-HepB) vaccine. Similarly, the risk of injection-site abscess would show no statistically significant difference between children received first aliquot given from a two-dose PCV-10 vaccine vial and those received the second aliquot from the same vial.

## Subjects and Methods

The study was conducted in four woredas (districts) located in four regions of the country that have existing University based Health and Demographic Surveillance System (HDSS). These HDSS are Kersa from Haramya University, Eastern Hararghe, Oromia Region; Dabat from the University of Gondar, North Gondar Zone, Amhara region; Arbaminch Zuria from Arba Minch Univerisy, Southern Nations Nationality and People Region; and Kiliete Awlaelo from Mekelle University, Eastern Tigray Zone, Tigray Region. For the purpose of census and other statistical data collection, kebeles (smallest administration units) are further divided into enumeration areas (EA). At all levels there are personnel responsible for providing services. At the kebele level, there are two Health Extension Workers (HEWs) responsible for organizing preventive services and maintain household records that are routinely updated annually. At the EA level, there are several community volunteers who assist the HEWs.

A longitudinal population-based multi-site observational study was conducted between September 2011 and October 2012. Data on vaccines received and adverse events following immunization, such as injection-site abscess, shock or death, were collected systematically and prospectively. Eligible populations for the study were children that are eligible to receive routine immunization at vaccination centres in the study area. The specific inclusion criteria include: resident of selected HDSS districts, provision of informed consent and age <1 year. For children who fulfill the inclusion criteria the only exclusion criterion for the study was receiving concomitant experimental vaccine/intervention.

The sample size calculation was made based on the following assumptions: detecting a relative risk of 2.0, baseline incidence of abscess formation following vaccination of 0.001, α = 0.01, power of 80%, and intera class correlation (ICC) = 0.4. Accordingly, approximately a minimum of 16, 500 vaccinated children were needed. Considering a 15% non-response and drop-out rate, the final sample size required for the study was approximately 19, 500 vaccinated children, i.e. 39,000 vaccine visits/episodes (assuming an average of two visits per child) over the study period.

Data were collected using various data capture systems that were based at vaccination centers, households and clinics.

### Enumeration and Identification of Eligible Children

The study began by conducting a complete registration of the children under the age of one year through house-to-house survey in all the study sites to create a database of eligible study population. Community interviewers initially visited all households in the selected study sites to identify and record all children under the age of one year. At this visit a photo ID with unique identification number was issued to mothers of eligible infants.

### Vaccination Center Surveillance

Vaccination information was collected using an improved vaccination card that has space to record the time and date of vaccination as well as the aliquot number (whether the dose was the first or second from the vial) and the injection site (whether it was on the left or right thigh). Designated health workers at vaccination centres administered routine EPI vaccines to eligible infants as per the guidelines provided by the FMOH. Accordingly, PCV injections should be given on the right thigh and Pentavalent injections should be given on the left thigh. Mothers enrolled in the study were encouraged to carry the photo ID produced by the study when seeking immunization services. Vaccines given to eligible children are immediately recorded on the improved child vaccination card and on the registration book at the facility. At the time of vaccination health workers advised parents to return to the health facility if AEFI such as an injection-site abscess is observed. Clinical mentors were trained to use consistent case identification between individuals and before and after identify abscess. Children identified during home follow up with an injection-site abscess following immunization with pentavalent vaccine or PCV-10 vaccine were considered suspect case of AEFI and were investigated by trained clinical mentors. When injection-site abscess was confirmed a digital photograph of the affected limb was obtained as a permanent record of the event.

### Household-based Surveillance

A household-based surveillance was the key component of the study. When registered children receive vaccine, follow up is initiated to observe whether the child develops AEFI or not. Community interviewers were trained to observe and screen for injection-site abscesses using uniform follow up visit forms. The community interviewers immediately notify suspect cases to the clinical mentor of the study. The clinical mentor visits the reported child immediately and if confirm the presence of abscess the child is immediately referred to the nearest referral health facility for further evaluation and treatment. If the AEFI is death, verbal autopsy was done to determine the cause of death using standardized verbal autopsy procedure after the traditional mourning period, which is about 45 days, to obtain additional information surrounding the death but not with the assumption of getting a definitive cause of death.

### Hospital-based Surveillance

A system to capture information on all hospitalized children that are eligible for the study was established in collaboration with the local and referral health facilities. The hospital surveillance was established to identify children hospitalized due to shock/severe illness following immunization. The study established this procedure before the introduction of PCV-10 vaccine in the locality. The clinical mentor visited health facilities weekly to inquire about admitted suspect cases or when AEFI is notified. All the data from the different sources were linked using a unique identification number.

Four different types of form were used to capture the required data. The first was *household registration form*: The registration form collects information about the household and the demographic characteristics of the eligible children. The household registrations were updated every two months by visiting all households in the study areas. The second was *Vaccination Card*: an addendum card to existing vaccination card was implemented in all vaccination centres in the study areas to collect information on the time and date of vaccination, type of aliquot the child received, injection site, and other vaccinations provided during the same session. The modified vaccination card was an attachment to existing immunization card so that any previous history of vaccination can also be tracked. The third was *Household follow up form*: community interviewers’ use this form for following vaccinated children at home at 48 hours and on the 7th day. The last form was *AEFI Event Form*: this form is completed for all children suspect for AEFI and has two sections. The first part that identifies the child was filled by the community interviewers and the second part was completed by the clinical mentor who examined the child AEFI events. Any AEFI confirmed by the clinical mentor was promptly reported. Digital photograph was obtained of the affected limb as a permanent record of the event and stored without personal identifying image or information.

To ensure the quality of data an extensive supervision scheme was put in place for all sources of data. Each level performed as per standard procedure and reported any irregularities immediately. Researchers themselves did extensive field supervision on a regular basis. Training manual was prepared and training was given by survey experts and clinicians for five days to all site researchers and field workers to standardize procedures. The training included basic principles of interviewing and surveillance, detecting and reporting AEFIs, follow up procedures, ethical conduct of research, and referral procedures.

Data from each source were cleaned, standardised, collated and merged (on personal ID number) to create a database of events, specific to each site, which provides temporal links between immunization events and potential adverse events. Data entry was done immediately after the second follow up (7th day) was completed for the vaccinated children. Data were examined by the data manger for consistency and data validation procedures were also integrated with data entry system to minimize errors during data entry.

The specific study objectives directed the data analysis. The major endpoints were injection site abscess and death. Comparison of the risk of injection site abscess on the left and right thigh to detect difference in abscess risk between PCV and Penta injections was done by computing the Relative Risk along with its 95% Confidence Interval. Comparison of the risk of injection site abscess among children receiving the first aliquot and second aliquot of PCV was made to detect difference in abscess risk between the two aliquots. Deaths were described based on the household surveillance and verbal autopsy.

### Ethics Statement

The study was reviewed and approval by the National Ethical Review Board of Ethiopia. The study in itself did not introduce any new procedure in the immunization programme. There were no risks associated with the research as it mainly did interview and follow by observation. Written informed consent was obtained from the mother/guardian of vaccinated children. Confidentiality was maintained by using unique identifiers. Children identified with AEFI were immediately referred and transported to the nearest health facility for appropriate case management.

## Results

A total of 55,268 vaccination episodes were observed during the one year study period. Of which 28,321(51.2%) were male and 26,947(48.8%) were female. Majorities (51.6%) were under two months old and only 3.9% were older than ten months (Table 1). The study observed 55,268 PCV, and 37,480 Pentavalent (DPT-Hep.B-Hib) vaccination episodes during the one year study period, which was higher than the sample size planned at the design stage. All children who received Pentavalent received PCV vaccination at the same time, while a total of 17,788 received only PCV vaccination. Of the total 55,268 PCV vaccinations 22,067 were PCV1, 18,718 PCV2, and 14,483 PCV3. Of the total 37,480 Pentavalent (DPT-Hep.B-Hib) vaccinations 12,912 were Penta1, 12,776 Penta2, and 11,792 Penta3 (Table 2). All vaccinated children in the study sites were visited at home at 48 hours and on the 7th day following vaccination by the designated field workers.

**Table 1:** Distribution of children received PCV Vaccination by age, sex and study site, Ethiopia, 2011/2012.

Study sites	Male					Female				
	Age in Months					Age in Months				
	0–2 M	3–5 M	6–9 M	10–12 M	Total	0–2 M	3–5 M	6–9 M	10–12 M	Total
Arbaminch	4620	1863	1272	156	**7911**	4259	1634	1232	137	**7262**
Dabat	2113	1085	965	187	**4350**	2110	1030	908	209	**4257**
Kersa	3572	2623	2236	420	**8851**	3585	2504	2061	317	**8467**
Kilite Awlaelo	4224	1060	1550	375	**7209**	4027	1094	1473	367	**6961**
Total	14529	6631	6023	1138	**28321**	13981	6262	5674	1030	**26947**

**Table 2:** The number of children received PCV and Pentavalent (DPT-Hep.B-Hib) Vaccination, Ethiopia, 2011/2012.

Study sites	PCV Vaccinated				Penta Vaccinated			
	PCV_1_	PCV_2_	PCV_3_	Total	Penta_1_	Penta_2_	Penta_3_	Total
Arbaminch	6246	5124	3803	**15173**	3238	3315	2838	**9391**
Dabat	3681	2928	1998	**8607**	2043	1974	1552	**5569**
Kersa	6839	5883	4596	**17318**	4759	4759	4953	**14471**
Kilite Awlaelo	5301	4783	4086	**14170**	2872	2728	2449	**8049**
Total	22067	18718	14483	**55268**	12912	12776	11792	**37480**

In all study sites, children who received the first aliquot (53.4%) of the PCV were slightly higher than those who received the second aliquot (46.6%). This is anticipated as health workers are expected to discard the 2nd aliquot if not used within 6 hours. The proportion of the first and second aliquot PCV vaccine offered to eligible children is similar across the four study sites (Table 3).

**Table 3:** The distribution of PCV aliquot given to children enrolled into the study, Ethiopia, 2011/2012.

Study sites	PCV Injection		Total
	1^st^ aliquot	2^nd^ aliquot	
Arbaminch	8113 (53.5%)	7051 (46.5%)	15164
Dabat	4837 (56.2%)	3769 (43.8%)	8606
Kersa	9166 (53.0%)	8130 (47.0%)	17296
Kilite Awlaelo	7378 (52.1%)	6785 (47.9%)	14163
Total	**29494 (53.4%)**	**25735 (46.6%)**	55229

Overall 99.6% of the PCV injections were given on the right thigh and 98.6% of the Pentavalent injections were given on the left thigh as recommended (Table 4). Oversight regarding the correct administration of the vaccine on the recommended side of thigh occurred only when a single vaccine is provided to the child.

**Table 4:** The distribution of the site of PCV injection among the study children, Ethiopia, 2011/2012.

Study sites	PCV vaccination		Penta Vaccination	
	Right thigh	Left thigh	Right thigh	Left thigh
Arbaminch Zuria	15120 (99.7%)	50 (0.3%)	249 (2.6%)	9160 (97.4%)
Dabat	8594 (99.8%)	13 (0.2%)	40 (0.7%)	5525 (98.3%)
Kersa	17131 (99.4%)	111 (0.6%)	162 (1.1%)	14448 (98.9%)
Kilite Awlaelo	14145 (99.8%)	22 (0.2%)	73 (0.9%)	7977 (99.1%)
**Total**	**54990 (99.6%)**	**196 (0.4%)**	**524 (1.4%)**	**37110 (98.6%)**

A total of 19 adverse events following immunization were observed during the follow up period; up to 7 days following immunization. Of which 10 were abscess and 9 were death. Seven of the ten abscess cases received only PCV vaccine and the rest three received both PCV and Pentavalent at the same time. All abscess cases were treated at health facilities and all fully recovered following appropriate treatment. Among the total 19 AEFIs, 11 of them were male and 8 were female. Moreover, 14 of them were under 6 months old and the rest 5 were 7–12 months old (Table 5).

**Table 5:** Adverse Events Following Immunization reported from each study site by sex and age, Ethiopia, 2011/2012.

	Type of AEFI										
	Abscess					Death					
Sex	Male		Female			Male		Female			Total AEFI
Age in Months	0–6 M	7–12 M	0–6 M	7–12 M	Total Abscess	0–6 M	7–12 M	0–6 M	7–12 M	Total Death	
Arbaminch	2	1	0	2	5	0	0	0	0	0	5
Dabat	1	0	0	0	1	2	1	1	0	4	5
Kersa	0	0	0	0	0	1	0	1	1	3	3
Kilite Awlaelo	3	0	1	0	4	0	0	2	0	2	6
**Total**	**6**	**1**	**1**	**2**	**10**	**3**	**1**	**4**	**1**	**9**	**19**

**Figure 1:**
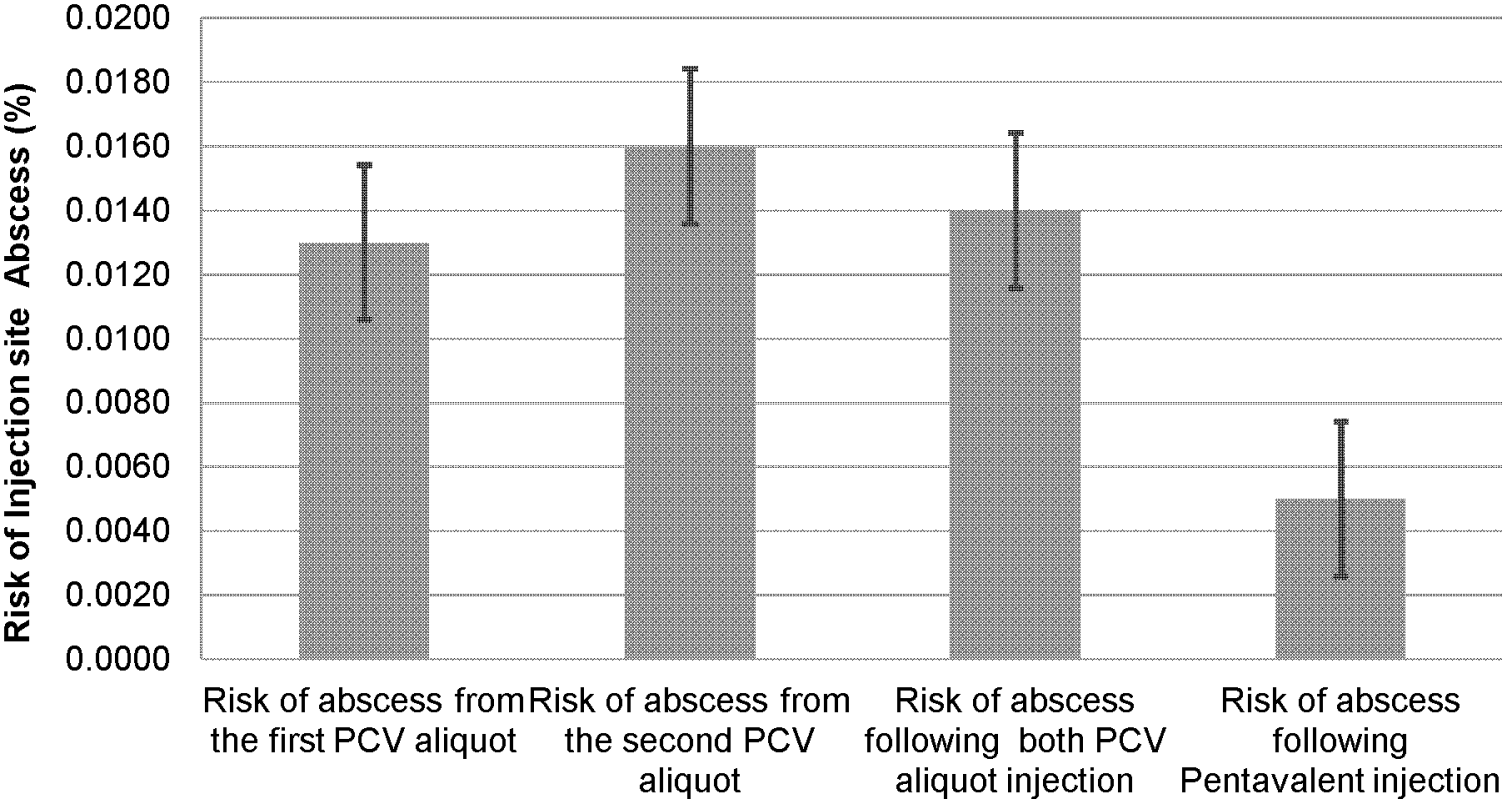
Risk of Injection site abscess for PVC-10 and Pentavelent Vaccinations, Ethiopia, 2011/2012. Key: Risk of Injection site Abscess in % with 95% Confidence Interval.

**Figure 2:**
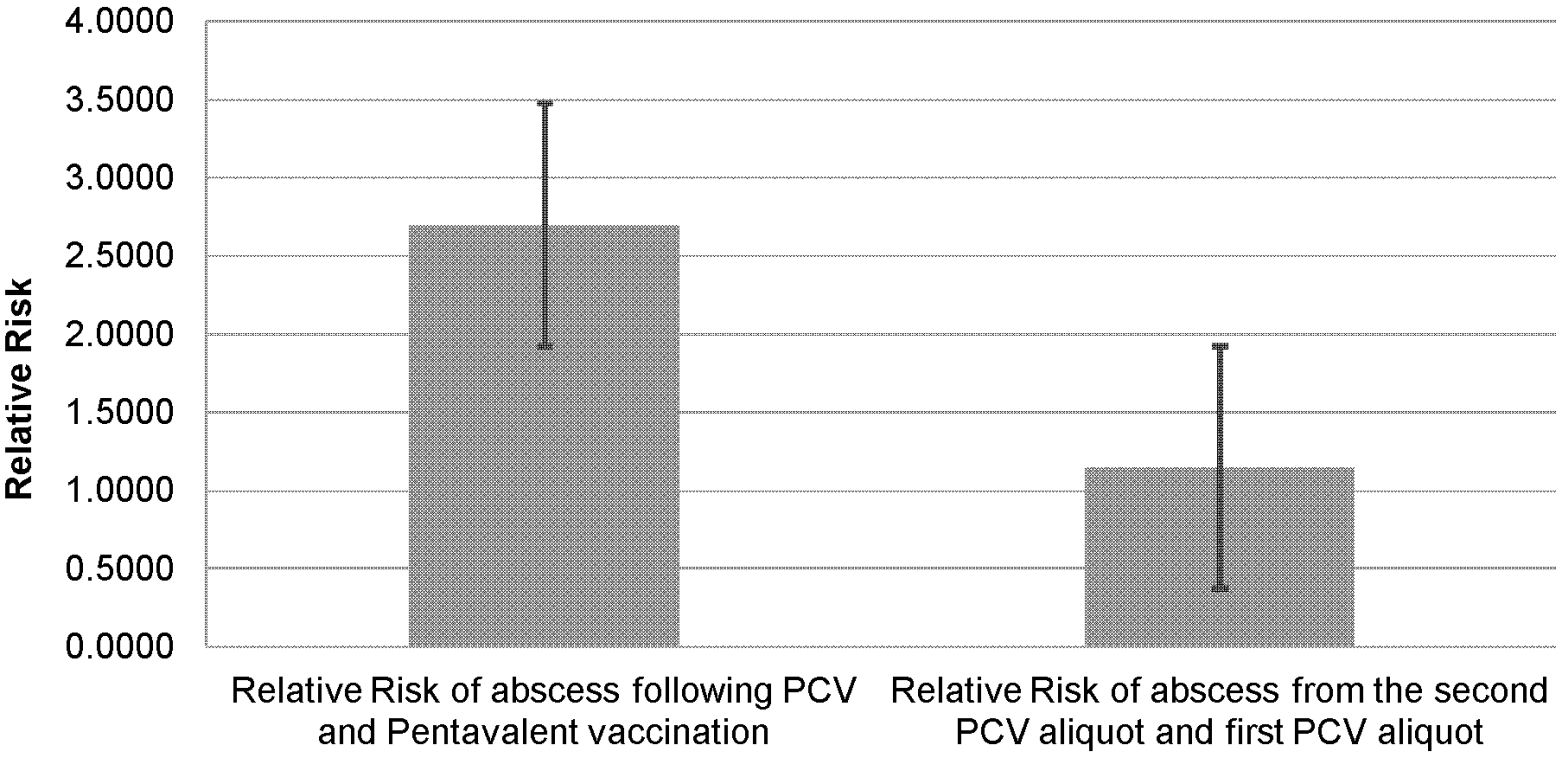
The relative risks for Injection site abscess for PVC-10 compared to Pentavelent and for PCV-10 first and second aliquot Vaccinations, Ethiopia, 2011/2012. Key: Relative Risk with 95% Confidence Interval.

As shown in Figure 1 and 2, no statistically significant increased risk of adverse events was observed between PCV and Pentavalent (DPT-Hep.B-Hib) injection sites (RR = 2.7; 95% CI 0.576–12.770; P = 0.204) as well as between the first and second aliquot of PCV injections (RR = 1.15; 95% CI 0.287–4.582; P = 0.851). The total number of deaths observed during the one year surveillance period was 9. All deceased children, except 1, received at least two different vaccines at the same time. Four of the deaths were reported from Dabat, 3 from Kersa and 2 from Kilite Awlaelo. No death was reported from Arbaminch. Four of the deaths were male and the rest 5 were female. Seven of the nine deceased children were younger than six months. Only two of the deceased children were age seven and eleven months at the time of death.

## Discussions

The risk of injection site abscess was comparable in children who received PCV-10 vaccine from a two-dose vial without preservative up to 7 days following vaccination compared to those who received pentavalent vaccine. The study also documented that there is no increased risk of injection site abscess in children who received the second aliquot of PCV-10 vaccine up to 7 days following vaccination compared to those who received the first aliquot.

Improper immunization practice with a two-dose preservative-free vial could potentially result in microbial contamination, non-sterile injections, and adverse events. Cognizant of such risks, the Federal Ministry of Health of Ethiopia in partnerships with key stakeholders conducted extensive training to familiarize the immunization staff at all levels about the necessary procedures on the proper use of the two-dose preservative-free vial. This is believed to have prevented any potential risk during the introduction of the new pneumonia vaccine in Ethiopia.

This population based longitudinal study was necessary to overcome the challenges in establishing the temporal relationship between a suspected AEFI and the date of vaccine administration. The study established an extensive network of vaccinators, community interviewers and clinical mentors to gather detailed information about the timing of vaccination and development of AEFI. Thus, this is one of the systematic studies conducted in large scale on immunization in Ethiopia that minimize recall and ascertainment biases. Another very critical aspect of this study was the control of potential confounders. Since the risk of adverse effects following immunization for PCV and Penta were observed in the same child there was no need to utilize other confounding control strategies.

The study managed to enroll more than the expected number of children into the study. That was possible because large population base and the extra efforts made by the study teams in creating awareness about the importance of immunization in general during their home visits. The sensitization increase immunization coverage and allowed the study to observe more children than originally anticipated. The increased sample size in turn increased the power of the study to detect differences, if there were any.

As the number of deaths is very small further analysis to determine the mortality risk difference was not possible, which was a recognized limitation at the design stage. Verbal autopsy was done to describe the cause of death suggested a probable vaccine related cause only for one of the deaths. Determining a definitive cause of death for any of the deaths was not possible because of lack of facilities in rural Ethiopia; again a recognized limitation at the design stage.

We conclude that if properly used as per the instruction of the manufacturers to eligible children in the routine immunization, preservative free two-dose vaccine vials cause no excess risk of Adverse Events Following Immunization.
